# Context-dependent concurrent adaptation to static and moving targets

**DOI:** 10.1371/journal.pone.0192476

**Published:** 2018-02-08

**Authors:** Maria N. Ayala, Denise Y. P. Henriques

**Affiliations:** 1 Department of Psychology, York University, Toronto, Ontario, Canada; 2 Centre for Vision Research, York University, Toronto, Ontario, Canada; 3 Department of Kinesiology and Health Sciences, York University, Toronto, Ontario, Canada; University of Exeter, UNITED KINGDOM

## Abstract

Is the neural control of movements towards moving targets independent to that of static targets? In the following experiments, we used a visuomotor rotation adaptation paradigm to examine the extent to which adapting arm movements to static targets generalize to that of moving targets (i.e. pursuit or tracking). In the first and second experiments, we showed that adaptation to perturbed tracking movements generalizes to reaching movements; reach aftereffects following perturbed tracking were about half the size (≈9°) of those produced following reach training (≈ 19°). Given these findings, in the final experiment we associated opposing perturbations (-30° and +30°) with either reaching or tracking movements and presented them within the same experimental block to determine whether these contexts allow for dual adaptation. We found that the group that experienced opposing perturbations was able to reduce both reaching and tracking errors, as well as produce reach aftereffects following dual training of ≈7°, which were substantially smaller than those produced when reach training was not concurrent with tracking training. This reduction in reach aftereffects is consistent with the extent of the interference from tracking training as measured by the reach aftereffects produced when only that condition was performed. These results suggest partial, but not complete, overlap in the learning processes involved in the acquisition of tracking and reaching movements.

## Introduction

In order to adapt and acquire new motor skills, the motor system must learn novel relationships between motor commands and their subsequent sensory consequences. The formation of these novel relationships (or “mappings”) allows us to maintain accurate movements despite having to switch from one behavioral or environmental context to another. To determine what affords the motor system this flexibility, we can associate specific contexts with distinct mappings and observe how this affects motor learning. When no context is provided, it is extremely difficult to adapt to opposing visuomotor maps [1,2]. However, when suitable contextual cues are associated with two or more distinct mappings, “dual adaptation” has been found to occur across different types of sensorimotor transformations including lateral prism shifts (e.g. [3]), force-fields (e.g. [4,5]), and visuomotor rotations (e.g. [6,7]). While a range of contextual cues have been examined, it remains unknown whether distinct types of movement can facilitate dual learning. Here, we explore the extent to which tracking movements (towards moving targets) generalize to reaching movements (towards static targets), and whether the motor system is able to distinguish and retrieve opposing visuomotor maps using these distinct movement types as contextual cues.

The current literature suggests that contextual cues related to the state of the end-effector best facilitate dual adaptation. In force-field adaptation tasks, it appears that visual cues unrelated to the state of the arm such as target, cursor, or background colour yield no changes in compensatory forces [4,8], while other cues that rely more on proprioceptive feedback such as workspace locations (i.e. divergent reach directions) seem to be more effective at facilitating dual learning [5,9–12]. In visuomotor adaptation tasks, dual adaptation has been shown to occur when opposing visuomotor maps were each associated with divergent starting locations (which elicited distinct arm postures), different hands, as well distinct postures of the hand or the body, to name a few [7,13,14]. Altogether, these findings from both force-field and visuomotor adaptation paradigms suggest that sensory information related to the arm provide the most useful context for allowing for concurrent motor learning. Here, we investigate another possible parameter related to the state of the arm that may facilitate dual learning: movement type.

Current models which explain the role of context in motor control suggest that distinct neural populations may be involved depending on the context variant [15,16]. Indeed, in recent theories of limb control, neural activity in the cortex is thought to reflect a dynamical system in which a preparatory state has a mechanistic role for the upcoming movement generation activity [17,18]. This has been supported by neurophysiological findings by Ames and colleagues (2014) in a movement delay and target switch task. They found that when there is no movement delay during a target switch condition (i.e. an action requiring a new motor plan), a new preparatory state is achieved that takes a parallel and separate path through the neural state space [17]. These findings could potentially explain the role of context and movement planning in concurrent learning, where distinct motor plans are required given a specific context. Indeed, after discovering the role of follow-through movements in adapting to opposing force-field perturbations, Howard and colleagues postulated the idea that different contexts might engage separate neural populations or perhaps simply alter the preparatory state [19]. More recently, Sheahan and colleagues (2016) showed that the planning of movements is a fundamental component in concurrent learning of opposing force-field perturbations. In their experiments, the planning component was isolated by having participants adapt to opposing force-field perturbations that were each predicted by a secondary target that either appears (i.e. execute secondary movement) or disappears mid-movement (i.e. do not execute secondary movement). They found that planning a secondary movement even without its execution allows for concurrent adaptation of opposing force-fields, even to the same extent as having always executed the planned movement [20]. These findings emphasize the importance of motor planning and the relevant movement parameters involved in dual adaptation.

While our aim is to investigate the cues necessary for concurrent adaptation within the same training block, interference studies that look specifically at task-dependent learning may also offer some insight as to what possible cues allow for dual adaptation. A key finding is that task dissimilarity seems to reduce interference experienced when learning opposing perturbations in a series of interference tasks [21]. In one experiment by Tong and Flanagan [21], participants reached to a clockwise (CW) rotation, followed by exposure to an opposing counter-clockwise (CCW) rotation when doing either the same reaching task or a different arm movement task (figure-eight drawing or continuous random tracking). Firstly, participants were able to adapt serially to the successive CW and CCW rotations, regardless of the task type. However, on retest of the reaching task, those who adapted to opposing rotations for the same task experienced the most interference while those who experienced opposing rotations for different tasks performed similarly as controls [21]. Their findings show that two different tasks can be learned in close succession with very little interference. Similarly, a set of recent ABA studies by Morehead and colleagues [22] looked specifically at transfer across different tasks and found the greatest amount of generalization when the training and test context were most congruent. Due to the constraints of the serial ABA interference paradigm, we are unable to infer how different tasks may affect each other (i.e., how tracking adaptation affects reaching adaptation), and ultimately, how both can be learned concurrently. These provide the insight that it is possible to learn a series of related tasks performed on separate blocks of training (i.e. ABA paradigm), but do not necessarily reflect whether they can be learned within the same block. To this end, we implement a concurrent learning paradigm in the present experiments to challenge the motor system to form and interchangeably use two distinct visuomotor mappings.

In addition, while both Tong & Flanagan [21] and Morehead et al. [22] suggest that the environmental context is conducive to reducing interference, they do not allow us to infer specifically which aspects of the environment allow for this. This is because different tasks may also require different movement parameters that may have unique and independent contributions in facilitating dual adaptation. For instance, Tong & Flanagan [21] found no interference when a different task was associated with the opposing interfering task that required movements in different directions, velocities, and task endpoints suggesting that subjects were planning movements for a completely different workspace as point-to-point reaching. Thus, these differences suggest that each task required a different movement type as well as an entirely distinct movement path (i.e. radial reaching movements vs. tracking movements in pursuit of an erratically-moving target). On the other hand, in their series of serial ABA experiments, Morehead et al. [22] used a gradually-introduced rotation under different training tasks including point-to-point reaching and random pursuit movements amongst others. The reach aftereffects produced following center-out and target-to-target reach training were the largest, presumably due to the similarity of the training and test learning contexts, while the least amount of transfer occurred for random movements. Using a laterally-displaced cursor, Simani et al. [23] found that track training of randomly-moving targets led to reach aftereffects and tracking aftereffects of similar magnitude. The cause of the positive transfer in Morehead et al. [22] and Simani et al. [23] and the lack of interference in Tong & Flanagan [21] are not clear, however, since certain movement types may require different movement directions which, on its own, have been shown to be successful at facilitating dual adaptation [6,10]. In a similar experiment, gradual introduction of the perturbation may also exert some effect as it has been shown that dual adaptation is possible when both rotations are gradually introduced [24]. Thus, to eliminate these possibilities and isolate the role of different movement types, we introduced the perturbations abruptly while holding movement and task endpoints (i.e. target locations) constant across perturbations. As a result, both reaching and tracking tasks require very similar outward movements with only the type of movement serving as the uniquely contributing contextual cue.

The key objective of the following experiments was to determine whether adaptation to a perturbation with distinct movements generalize to other movement types, specifically whether tracking movements towards moving targets generalize to reaching movements towards static targets, and further, to see if these distinct movement types can facilitate dual adaptation. First, we examined whether adaptation to a rotated tracking task produces significant learning and reach aftereffects. One group of participants made out-and-back reaches to static targets with a 30° CW-rotated cursor while a different group tracked a moving target with a 30° CCW-rotated cursor. If adapted tracking movements do not entirely generalize to reaching movements (seen via reach aftereffects following adaptation to a perturbed tracking task), it is possible that distinct movement types can facilitate adaptation to two opposing visuomotor rotations. To this end, we completed another experiment in which we used a concurrent (dual adaptation) interference paradigm to investigate the efficacy of different movement types in facilitating the acquisition of opposing visuomotor maps. In this third group of participants, both reaching (associated with a 30° CW-rotated cursor) and tracking (associated with a 30° CCW-rotated cursor) trials were interleaved within the same experimental block. This task is distinct from the typical interference task such that adaptation to both visuomotor rotations can be analyzed as they are acquired simultaneously.

## Methods

### Participants

Fifty-four participants (eighteen males and thirty-six females, 19.97 ± 2.44 years old, mean ± SD) were recruited and assigned to participate in the following experiments and were granted a bonus credit for an undergraduate psychology course. All participants were right-handed, had normal or corrected vision, and were naïve to the purpose of the experiment. All participants provided written, informed consent. Procedures were approved by York University’s Human Participant Review Committee and were in accordance with the declaration of Helsinki. Due to arm discomfort, two participants chose to discontinue and were excluded from the experiments.

### Apparatus

**Fig 1:**
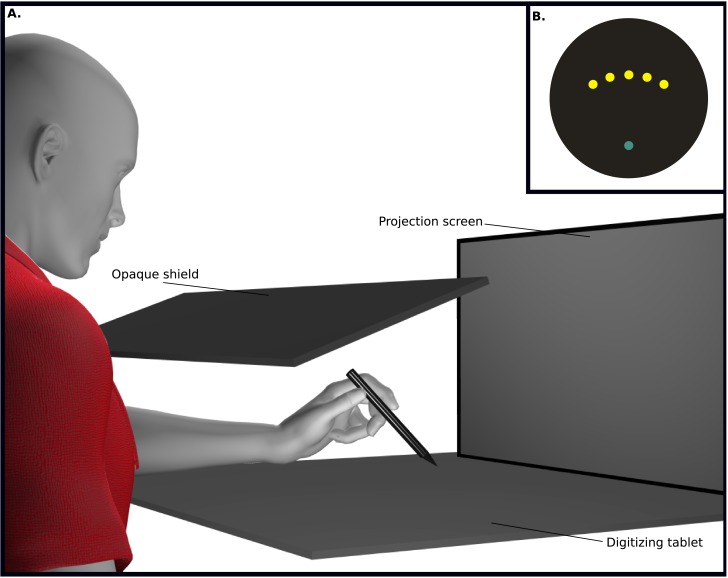
Experimental apparatus and display of target. Stimuli were projected (using a projector) on a vertically positioned screen approximately 60 cm away from the tablet. Participants reached or tracked targets on a digitizing tablet (A) using a handheld stylus on the horizontal plane while observing a projected image of the targets and cursor on a circular-edged vertical screen. The light blue circle represents the home position (B). The five yellow circles represent the five possible locations for the target (60°, 75°, 90°, 105°, 120°) which were the same across reaching and tracking trials. (A) is depicted as mirror image.

Participants sat on an adjustable chair facing a digitizing tablet (Wacom Intuos3, 12” x 12” surface, resolution of 5080 lines/inch, sampled at 50 Hz) and screen. The tablet was placed at waist level so that hand movements were made along the horizontal plane (See Fig 1 for detail). An Epson 3LCD projector rear-projected an image onto the screen located approximately 60 cm from the tablet workspace. To prevent participants from observing their arm movements, an opaque shield was placed above the tablet work surface [6,25,26]. All tasks involved five radially-spaced targets located at 60°, 75°, 90°, 105°, and 120°. All targets began at a common starting point located 12 cm away from the home position (during the reaching trials), or beginning at the home position and landing at the same final target locations (during tracking trials). Participants acquired the targets (1.5 cm in diameter) using a hand-held stylus that they moved across the surface of the tablet, moving a cursor (1 cm in diameter) on the screen (Fig 1, inset). The relationship between hand and cursor was similar to using a desktop computer; movements were made with a 1:1 ratio.

### General procedure

**Fig 2:**
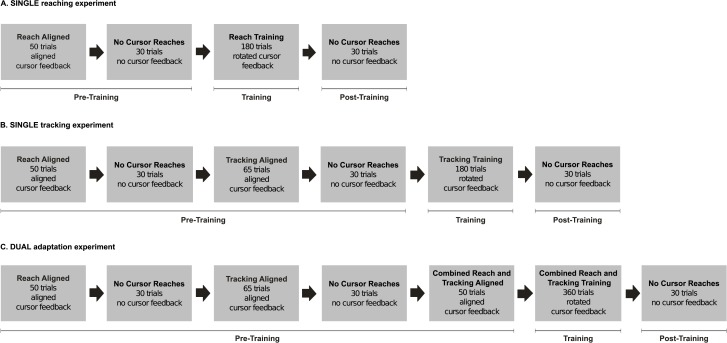
Session sequence. Session sequence for the SINGLE reaching experiment (A), the SINGLE tracking experiment (B), and the DUAL adaptation experiment (C). For the SINGLE experiments, only one distortion was experienced throughout training: counter-clockwise (CCW) for the SINGLE tracking experiment, and clockwise (CW) for the SINGLE reaching experiment. The DUAL adaptation experimental group experienced both reaching and tracking trials, which were associated with CW and CCW rotations (i.e. with the same association as in SINGLE reaching and tracking experiments), respectively. Critically, no-cursor trials were identical across all conditions—there was no visual feedback of the cursor and thus were all open-loop reaches.

In the first experiment (henceforth referred to as the “SINGLE reaching experiment”), we examined adaptation to a single visuomotor rotation while reaching to static targets (Fig 2A). This experiment served as a control group for the following two experiments. In the second experiment (henceforth referred to as the “SINGLE tracking experiment”), we examined adaptive tracking movements to a semi-predictable target trajectory when presented with a visuomotor rotation (Fig 2B). Additionally, we examined whether this adaptation to a rotated tracking task influences reach adaptation by looking at any present reach aftereffects following rotated tracking training. Reach aftereffects from the SINGLE reaching experiment served as the control for that of the SINGLE tracking experiment. Given the results of the SINGLE tracking experiment, we conducted another experiment (henceforth referred to as the “DUAL adaptation experiment”) where we investigated whether dual adaptation to tracking and reaching movements occurs when each type of movement was associated with distinct and opposing visuomotor rotations (Fig 2C). Tracking adaptation results from the SINGLE tracking experiment served as the control group for the tracking trials in the DUAL adaptation experiment. Reach adaptation results from the SINGLE reaching experiment served as the control group for the reaching trials in the DUAL adaptation experiment.

Depending on the trial type (TRACK or REACH trial), participants either tracked a moving target or reached toward a static target. TRACK and REACH trials were preceded by “T” and “R” across all experiments, respectively. Targets appeared 12 cm away from home position (REACH trials) or moved 12 cm from home position (TRACK trials) but all reached the same 5 possible final target locations of either 60°, 75°, 90°, 105° or 120°. All participants started at the same home position and were told to either make smooth and direct reaches to the target (REACH trials) or to follow the target as closely and accurately as possible (TRACK trials). For TRACK trials, the moving targets took 1500 ms to cover the 12 cm distance (8 cm per second) for all but the initial 15 baseline trials. This incremental change in overall movement time ensured that participants were initially accustomed to pursuing the target, and were able to adjust to a faster speed as the condition progressed. During baseline aligned (veridical) tracking trials in both the SINGLE tracking and DUAL adaptation experiments, the overall movement time of the target started off at 1800 ms at trial 1, and decreased by increments of 20 ms during each trial until it reached 1500 ms. By trial 16, the target movement time was always 1500 ms, including trial 1 of the rotated tracking conditions.

To aid and motivate the participants during TRACK trials, the colour of the target changed depending on the distance of the cursor from the target. The target appeared green when the cursor successfully overlapped with the target, yellow when it was 1 to 4 cm away, orange when it was 4 to 8 cm away and red when it was more than 8 cm away from the target. The home position appeared as a light blue circle on the screen (1.6 cm), and the cursor appeared as a smaller white circle (1 cm). Each target appeared individually in a pseudo-randomized order.

For trials involving visual feedback of the hand-cursor (also referred to as “closed-loop trials”), participants pursued a moving target, or reached towards a static target in order to complete the trial. For trials involving no visual cursor feedback (also referred to as “open-loop trials”), participants reached towards the target with their unseen cursor, and remained stationary for 500 ms until the home position appeared. Visual feedback of the hand-cursor appeared when participants were within a 2 cm radius of the home position. In order to facilitate the return to the home position during open-loop trials, a smiley-face represented the home position that changed in orientation depending on the position of the pen. We also placed a solid edge located just below the home position to help guide movement back to home position. Prior to no-cursor trials, participants were merely told that the cursor would not be visible. We did not include any specific instructions (i.e. whether or not to implement a strategy, as in [27]) since recent work by Werner and colleagues [28] as well as results from our lab [29] suggest that for small rotations, instructions for the no-cursor tasks do not affect the size of the reach aftereffect.

We did not measure tracking aftereffects due to the assumption that smooth tracking (i.e. maintaining the same speed as that produced during training) would require a visible cursor. Instead, we exclusively captured reach aftereffects for all three groups. This allowed us to test whether adaptation of tracking movements could transfer to reaching movements, and whether concurrent training of tracking and reaching movements while each was associated with an opposing perturbation would interfere (i.e. reduced reach aftereffect in the DUAL group).

For each experiment, participants completed pre-training, training, and post-training sessions. In both the SINGLE tracking experiment and the SINGLE reaching experiment, participants completed the task in approximately one hour. In the DUAL adaptation experiment, participants completed the task in approximately two hours. Participants were not informed about the presence or nature of the rotation at any point in the experiments. After the experiment, participants answered a series of questions to assess their awareness of the rotation and the experimental objectives.

### Experiment 1: SINGLE reaching experiment

Nineteen participants completed the SINGLE reaching experiment, which served as the control group for the reaching trials in the DUAL adaptation experiment. During training, these participants experienced a 30° CW rotation of the cursor (Fig 2A, box 3); this was congruent with the associated rotation and movement type in the DUAL adaptation experiment (Fig 2C, box 6).

#### Aligned training (Baseline measures)

The aim of the pre-training session was to obtain baseline performance and familiarize participants with the task. Participants reached for static targets 50 times with visual feedback of the aligned cursor, followed by 30 reaches with no visual feedback of the cursor (i.e. no-cursor) in order to record baseline open-loop reach errors (Fig 2A, boxes 1 & 2).

#### Rotated reach training (Adaptation) and post-training (Reach aftereffects)

The aim of training was to expose participants to a single visuomotor rotation in order to assess the learning rate and reach aftereffects to compare to the reaching conditions in the DUAL adaptation experiment. Participants reached 180 times with a 30° CW rotated cursor, followed by 30 no-cursor reaches (Fig 2A, boxes 3 & 4).

### Experiment 2: SINGLE tracking experiment

#### Aligned training (Baseline measures)

Seventeen participants completed the SINGLE tracking condition. During pre-training sessions, participants reached towards static targets (REACH trials), and tracked moving targets (TRACK trials), both with an aligned cursor (Fig 2B, boxes 1 & 3). The aim of the pre-training session was to familiarize participants with the tasks and capture baseline performance. During the aligned REACH training session, participants reached 50 times to static targets, followed by 30 no-cursor reaches to record baseline reach aftereffects. During the aligned tracking pre-training session, participants tracked moving targets 65 times, followed by 30 no-cursor reaches. The extra 15 trials for baseline tracking was to familiarize participants with the task; the target-movement duration began at 1800 ms but was gradually reduced to 1500 ms for the remaining trials and for all the rotated tracking trials.

#### Rotated tracking training (Adaptation) and post-training (After-effects)

Participants experienced a 30° CCW rotation during rotated training while tracking a moving target 180 times (Fig 2B, box 5). This was followed by 30 no-cursor reaches to assess generalization of what was learned during tracking trials towards reaching movements.

### Experiment 3: DUAL adaptation experiment

#### Aligned training (Baseline measures)

Sixteen participants completed the DUAL adaptation experiment. Like the SINGLE tracking experiment, DUAL participants reached 50 times with aligned visual feedback of the cursor (Fig 2C, box 1), followed by 30 no-cursor reaches. Then, participants tracked moving targets 65 times with an aligned cursor (Fig 2C, box 3), followed by 30 no-cursor reaches. Lastly, participants completed a condition combining both the reaching and tracking tasks. Participants either reached towards a static target 25 times or tracked a moving target 25 times with an aligned cursor (Fig 2C, box 5).

#### Dual training (Adaptation) and post-training (Reach aftereffects)

During rotated training in the DUAL adaptation experiment, participants experienced opposing visuomotor rotations, each associated with either TRACK or REACH trials, in order to determine whether dual adaptation can occur when cued by different types of movements. Participants in the DUAL group experienced both a 30° CW rotation (associated with REACH trials) and a 30° CCW rotation (associated with TRACK trials). Again, prior to each trial, participants saw a visual cue to indicate whether to expect a REACH or a TRACK trial (i.e. a “T” appeared before tracking trials and an “R” appeared before reaching trials). Participants completed 180 REACH and 180 TRACK trials (Fig 2C, box 6). REACH and TRACK trials were presented in a pseudo-randomized order such that participants encountered all five possible targets for each type of trial (and thus, rotation) before any target location was repeated. This was followed by 30 no-cursor reaches. Reaches during no-cursor trials were always made towards static targets due to the difficulty in acquiring a moving target without visual feedback, or controlling for tracking speed of the unseen hand.

### Data analysis

Cursor movement data was digitally smoothed using a first-order, low-pass Butterworth filter with a frequency cut-off of 2.5 Hz. Movement onset was fixed at 10% of peak velocity for REACH trials and 33% of peak velocity for TRACK trials. Tracking and reach adaptation to a visuomotor rotation can be assessed using a variety of dependent measures. For reaching tasks (including no-cursor trials), performance was quantified using “angular error at maximum velocity” which refers to the angular difference of the target and cursor at peak velocity relative to the home position. Due to the novelty of the semi-predictable tracking task, we reported several alternative performance measures as well as other movement time descriptors (described further below). For all our analyses involving blocks, we averaged across blocks of 5 trials in order to include a movement to each target.

#### Analysis of reaching adaptation (Reach training and no-cursor trials)

To compare reach adaptation between groups who received SINGLE or DUAL training, we compared angular reach errors across the first trial, the second block, and the final blocks of trials using a 3 (block) x 2 (group, SINGLE vs DUAL) mixed analysis of variance (ANOVA) followed by post-hoc t-tests with Bonferroni correction. In order to fully show the rapid learning occurring in the initial stages of learning, we chose to analyze the very first trial. Not only does blocking the initial stage of learning mask the rate of adaptation, these rapid trial-dependent changes will lead to a highly variable measure compared to those produced in later stages of adaptation. The overall difference between the first trial and the last block reflect the extent of learning, while the second block (relative to the first) provides a rough estimate of the learning rate. A one-way ANOVA (three levels: first trial, second block, final block) was used afterwards to show whether adaptation has occurred within each group. We also compared the performance between groups during the final block of rotated training to further show the magnitude of adaptation achieved.

Reach aftereffects refer to rotation-dependent, deviated reaching in the absence of visual feedback of the cursor. They were assessed by comparing the first block of trials after rotated-cursor training with the final block of trials after aligned-cursor training. To quantify reach aftereffects, we took the differences in no-cursor reach errors following rotated and aligned-baseline training. Then, to determine whether these reach aftereffects differed across the three groups, we ran a one-way ANOVA with three levels (SINGLE reaching group, SINGLE tracking group, DUAL group). Follow-up analyses using independent t-tests with Bonferroni correction revealed which pairs of groups had significantly differed in the magnitude of reach aftereffects.

#### Analysis of tracking adaptation (TRACK trials only)

We used several measures of tracking performance. Our main measure was the Root-mean-square-error (RMSE) which we calculated as follows:

$$RMSE\;=\;\sqrt{\sum_{i\;=\;1}^{N}{\left(\theta_{cursor}(i)-\;\theta_{target}(i)\right)}^{2}/N}$$

Where *θ*_*cursor*_(*i*) and *θ*_*target*_(*i*) represent the cursor and target positions in Cartesian co-ordinates at the *i*^th^ sample, respectively, and *N* is the total number of samples of the tracking trajectory in a trial. As participants adapted to the visuomotor rotation, we expected the RMSE to decrease, such that cursor-to-target tracking trajectories aligned over training trials. As we did with reach training, we assessed this adaptation while participants tracked a moving target by comparing the first trial, the second block, and the final block using a 3 (block) by 2 (group) mixed ANOVA, followed by post-hoc comparisons. These blocks allow us to assess the extent of adaptation, as well as the rate of learning. A one-way ANOVA (three levels: first trial, second block, final block) was used to show whether adaptation has occurred within each group. We also tested whether tracking performance during rotated-cursor training returned to baseline levels by comparing the final block of trials of rotated-cursor training with those for aligned-cursor training for each group using dependent t-tests. The assumed level of significance was *α* = .05, and post-hoc comparisons were Bonferroni-corrected.

#### Additional tracking descriptors (TRACK trials only)

To quantify overall tracking ability, we examined the change in the distance and time between the target and the cursor-movement onset (defined as the time when the cursor motion reached 33% of its peak velocity; i.e. tracking-cursor movement onset). Thus, we measured the time it took for the cursor to move in response to target motion (“target pursuit latency”) and their respective distance at cursor-movement onset (“cursor-to-target onset distance”). Aside from movement latency, we also analyzed overall cursor movement time from movement onset (passing the onset cutoff at 33% of maximum velocity) until offset (until the offset cutoff at 33% maximum velocity) using the same method as the main tracking measures. This overall cursor movement time is yet another descriptor of how well participants are able to match the target movement of 1500 ms (excluding familiarization trials where target movement is <1500 ms). We applied the same statistical analysis to these additional measures as we did to the main tracking measures.

To further quantify and illustrate the change in reaching and tracking errors across training, we fitted a single exponential function to all datasets across all blocks (of 5 trials) of training and averaged across participants, for each rotation and group using VEEL (http://veel.sourceforge.net/). The equation takes the form:

$$RD\;=\;{be}^{\left(-ax\right)}+c$$

Where *x* represents the block number, *a* the rate of learning, *c* the asymptotic level of performance, and *b* the scaling factor.

## Results

We examined whether visuomotor adaptation to tracking movements (towards moving targets) generalize to that of reaching movements (towards static targets) and further, whether these different types of movements were distinct enough to allow for dual adaptation to opposing perturbations. First, we tested whether participants can adapt tracking movements towards moving targets in response to a visuomotor perturbation and then immediately had them reach towards static targets and compared this to a group that only adapted their reaches to static targets. Next, we associated opposing perturbations to either reaching or tracking movements and presented them within the same experimental block.

### Reach adaptation (Performance during training)

**Fig 3:**
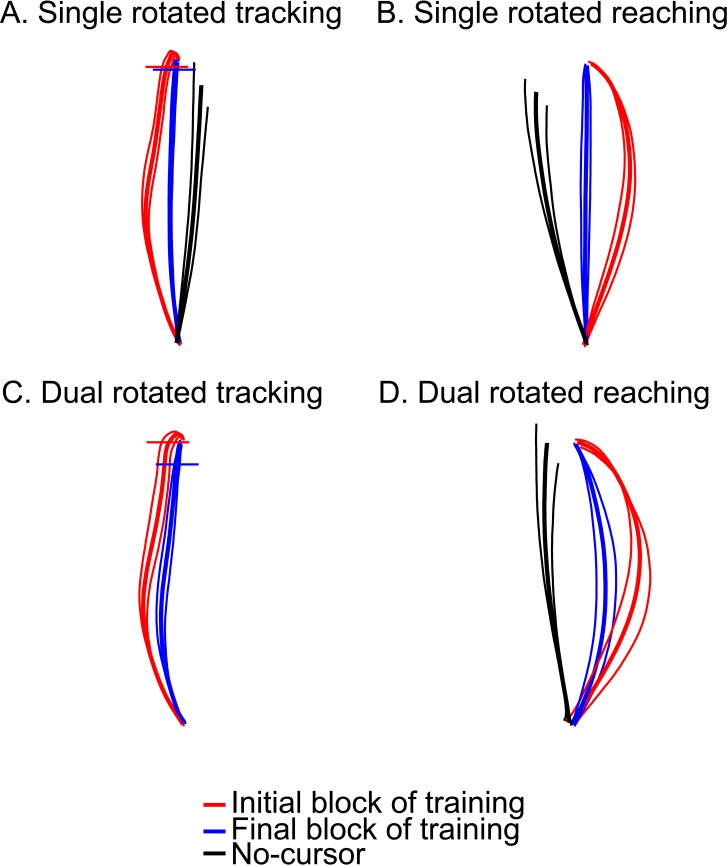
Average cursor trajectories for SINGLE and DUAL groups separated by rotations and collapsed across all target locations. Shown are mean hand paths for the SINGLE tracking group (A), the SINGLE reaching group (B), DUAL tracking trials (C), and DUAL reaching trials (D). Mean paths for the first five trials are in red and the last five trials in blue. The mean (central solid line), 95% confidence limits (two thin bordering lines) are plotted across all participants for each groups and rotation. Solid black lines represent mean paths for No-Cursor trials where there was no visual feedback of the cursor. There were no No-Cursor trials associated with the DUAL tracking trials as all No-Cursor trials are considered reaches (i.e. target is static). Horizontal lines for tracking trials depict the location of the cursor when the target halted at its final location.

Fig 3 illustrates mean hand path trajectories for each experiment and condition for the initial and final blocks bounded by 95% confidence intervals. As REACH trials (Fig 3B & 3D) were associated with a 30°CW rotation, participants compensated with reaches in the counterclockwise direction. Participants produced large, rotation-dependent reaching errors that reduced over training, exhibited by straighter trajectories that approach pre-training levels.

**Fig 4:**
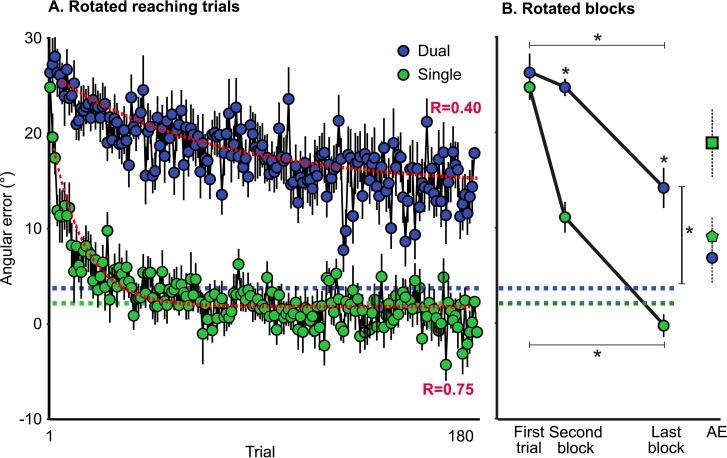
Angular reach error across rotated training for the SINGLE and DUAL groups for reaching trials only. Mean reach errors are depicted across all rotated training trials (A) and the initial trial, second block, and final block of 5 trials, in green for the SINGLE reaching-only group and in blue for the reaching trials for the DUAL group (B). Green and blue dashed lines represent baseline levels from aligned reaching conditions. Red dashed lines represent fitted exponential curves for reach deviations for the entire training session with the equation *RD = be*^*−ax*^*+c*. For the SINGLE group, *RD* = 16.72^−0.07x^ + 1.65, and for the DUAL group, *RD* = 11.17^−0.01x^ + 14.33. Single data points with dashed error bars in (B) represent normalized reach aftereffects (AE) for each group (SINGLE reach group represented by a square, SINGLE tracking group by a pentagon, and DUAL group by a circle). Error bars represent SEM.

To illustrate adaptation, Fig 4 shows the mean angular reaching errors produced by both the SINGLE group (green) and the DUAL group (blue) across all rotated-reaching trials (A) and for the first trial, the second block, and the final block (B). We fitted exponential curves to the blocked mean angular errors and show them in red dashed lines for both groups; each curve resembles a typical exponential curve associated with motor learning [30].

As evidenced by straighter reach trajectories (Fig 3) and fit to typical exponential learning curves (Fig 4), reaching errors significantly reduced across the first trial, the second block, and the final block, but this reduction differed significantly between SINGLE and DUAL groups (F(2,30) = 12.25, p <0.001). Nonetheless, we saw a significant reduction in error across training in the DUAL group (F(2,30) = 13.76, p<0.001) although this was smaller compared to that of the SINGLE group (F(2,36) = 116.21, p<0.001). To estimate the learning rate, we compared the second block of training trials between groups, and found that the SINGLE group was already showing a significantly larger reduction in error compared to the DUAL group (t(33) = -7.47, p <0.001). By the end of training, the DUAL group still made larger reaching errors compared to the SINGLE group (t(33 = -6.69, p<0.001). Thus, it appears that the DUAL group was able to show a significant reduction in error but not at the same rate or to the same extent as that of the SINGLE group. That is, movement type provided a viable context for each opposing visuomotor map such that less interference was experienced when both adapting to track a moving target and reach to a static target.

### Tracking adaptation (Performance during training and baseline)

**Fig 5:**
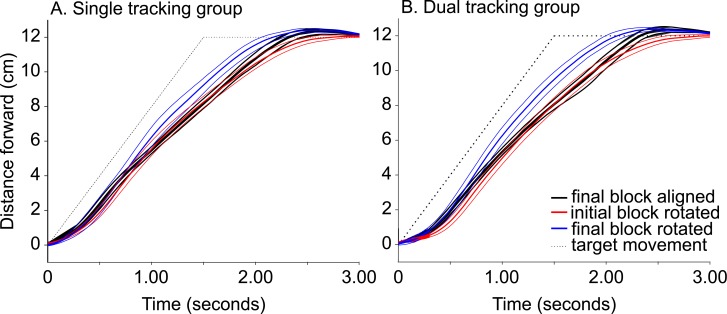
Mean cursor displacement. Mean cursor displacement across time for both aligned and rotated tracking trials for the SINGLE tracking group (A) and DUAL (B) tracking trials. Mean paths for the last five aligned tracking trials are depicted in black, while the first five rotated tracking trials are depicted in red and the last five rotated tracking trials in blue. The first block of the aligned trials was omitted as it largely overlaps with the final block of aligned trials. The mean (central solid line), 95% confidence limits (two bordering lines) are plotted across participants for each groups and conditions (aligned or rotated). The dashed line represents the semi-predictable path of the target with an overall movement time of 1500 ms.

Before exploring tracking adaptation (spatial accuracy), we first wanted to verify that participants were able to temporally pursue the moving target. Solid traces in Fig 5A and 5B show the distance the cursor moved in the direction of the target motion (shown in dotted trace) across time for the final block of training in both the aligned (black solid line) and rotated blocks (red solid line for the initial block and blue for the final block). For aligned training as well as the initial block of rotated-training, participants were reasonably able to keep up with the target, following only approximately a half-second (or a couple centimeters) behind. This suggests that introducing the rotation did not disrupt how closely participants were able to pursue the target, although it took longer to acquire the target site likely because of the direction-related correction due to the rotation. In other words, altering the visual feedback of the cursor did not disrupt the ability to pursue a moving target with regard to timing.

After confirming that the visuomotor rotation did not disrupt pursuit speed, we can now examine the effect of the perturbation on pursuit accuracy. This effect can be seen in Fig 3A and 3C, where the 30°CCW rotation (associated with tracking trials) led to large deviations in hand motion during the first block of training (red traces). Evidently, tracking movements became less deviated by the final block of tracking training (blue traces) for both the SINGLE (Fig 3A) and DUAL (Fig 3C) groups.

**Fig 6:**
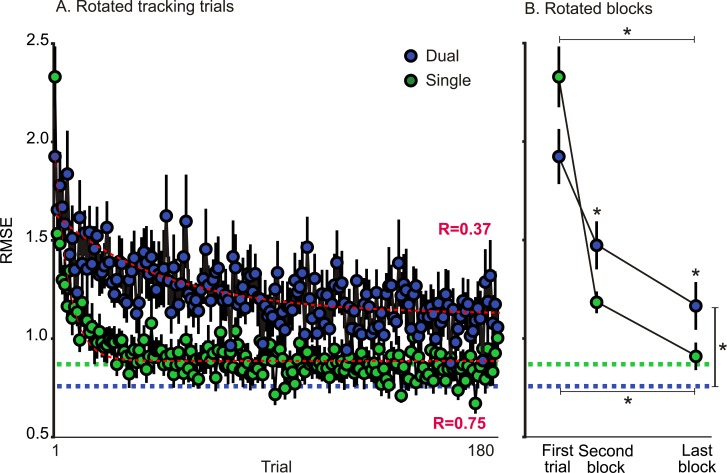
Root-mean-square-error (RMSE) across rotated TRACK training for the SINGLE and DUAL groups for tracking trials only. Mean RMSE are depicted across all rotated trials (A), and the initial trial, second block, and final blocks (of 5 trials) in green for the SINGLE tracking-only group and in blue for the tracking trials for the DUAL group (B). The DUAL group also completed aligned trials with both REACH and TRACK trials within the same experimental block (TRACK trials for this condition depicted in turquoise). Green and blue dashed lines represent baseline levels from aligned tracking conditions. Red dashed lines represent fitted exponential curves for tracking deviations for the entire training session with the equation *RD = be*^*−ax*^*+c*. For the SINGLE group, *RD* = -1.04^−0.18x^ -0.88, and for the DUAL group, *RD* = -0.51^−0.01x^ -1.12. Error bars represent SEM.

To assess adaptation during rotated tracking training, we measured tracking error as the accumulated difference between the moving cursor and target (RMSE). Tracking errors are plotted across trials in Fig 6A and across the first trial, the second block, and the final block in Fig 6B. Tracking errors from the first rotated trials were three times larger than those produced in the aligned tracking condition for both SINGLE and DUAL groups; we found no difference between groups at this point (t(31) = -0.47, p = 0.640). This initial trial was subsequently followed by a reduction in tracking error that varied in magnitude depending on the group (F(2,32) = 8.11, p<0.001). That is, while both tracking errors significantly decreased across training for the both the SINGLE (F(2,32) = 73.24, p<0.001) and DUAL (F(2,30) = 19.50,p<0.001) groups, the rate and amount of reduction differed. To estimate the learning rate, we compared the second block between groups and found faster learning already occurring early on for the SINGLE group compared to the DUAL group (t(31) = 2.34, p<0.05). By the final block, the SINGLE group had reduced their tracking error slightly more compared to the DUAL group (t(31) = 2.27, p<0.05). While final baseline levels did not vary between groups (Fig 6, horizontal dashed lines), only the SINGLE group was able to reduce their errors during rotated training to baseline levels (t(16) = 0.90, p = 0.384). Thus, as suggested by the performance of the DUAL group, it appears that associating opposing visuomotor maps with distinct types of movements reduced the amount of interference experienced when adapting to both perturbations concurrently.

### Additional tracking movement descriptors

As consistent with the results illustrated in Fig 5, described briefly above, overall cursor movement time for tracking trials was slightly over 2 s, and did not significantly reduce between the first and final blocks of aligned training (F(1,31) = 3.48, p = 0.072). However, introducing the rotation (for either group) lead to a transient increase in movement time for the first block of about 0.22 s (or ≈10% of the movement time) that returned to the baseline levels by the end of the block (F(1,31) = 0.24, p = 0.630). This pattern held for both SINGLE and DUAL training.

Cursor-to-target distance at onset significantly changed over time during rotated trials (F(2,62) = 17.06, p < .001) though this did not change as a function of group. This is likely due to the introduction of the novel rotations, which causes a large discrepancy in the initial stages of training but becomes smaller as participants adapt. For the DUAL condition specifically, cursor-to-target onset distance did not differ between the final block of aligned trials and the final block of rotated trials (F(1,15) = 1.04, p = 0.323). This suggests that the distance it initially takes participants to catch up to the moving target becomes uniform over time across conditions.

Target pursuit latency did not significantly change over time in any of the aligned or rotated blocks for both groups (see Fig 5). We also analyzed cursor movement time from target-movement onset until target-movement offset and found no significant change over time in any of the aligned or rotated conditions between groups. Thus, it appears that neither the time to pursuit nor the pursuit time changed across training.

### Reach aftereffects

**Fig 7:**
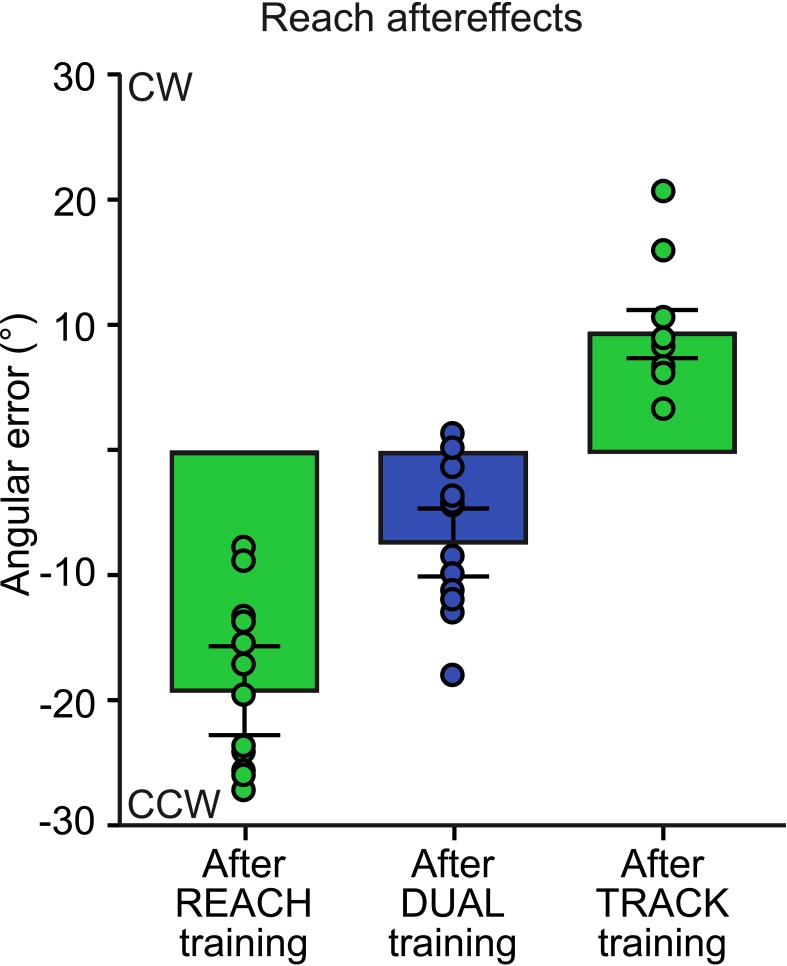
Reach aftereffects for SINGLE and DUAL groups. Mean angular error for the first block (of 5 trials) in No-Cursor trials following rotated training (subtracted by mean angular error of No-Cursor trials following aligned training for the first block of trials) are shown in green bars for SINGLE distortion groups and with a blue bar for the DUAL distortion group. Individual subject means are depicted in circles. Error bars represent SEM.

To illustrate reach aftereffects, the solid black lines in Fig 3 depict the mean cursor trajectory during No-Cursor reach trials following rotated training for all groups separated by rotations (or trial type, for the DUAL group). Fig 7 depicts the mean reach aftereffects for the SINGLE distortion reaching-only and tracking-only groups (both in green bars), and the DUAL distortion group. Following training with a perturbed cursor, the SINGLE reaching group demonstrated significant reach aftereffects in the expected (opposite) direction (t(13) = 10.66, p < .001). Likewise, following perturbed tracking training, the SINGLE tracking group demonstrated significant reach aftereffects, also in the expected direction for this opposite rotation (t(16) = -9.77, p < .001), although to a lesser extent compared to those produced by the SINGLE reaching group (t(29) = -4.96, p < .001). On average, reach aftereffects produced following only TRACK training were ~+9°, approximately half the size of those produced following REACH training (~-19°). This shows that adaptation to perturbed tracking movements generalizes to reaching movements, as adaptation effects transferred from one movement type (from training trials) to another during no-cursor trials. Altogether, these findings suggest that adaptation to a perturbed cursor when tracking a moving target generalizes to open-loop reaching, such that significant aftereffects manifest despite the difference in the type of movement.

Finally, following concurrent training where reach trials were associated with a CW rotation and track trials with a CCW rotation (DUAL group), participants produced significant reach aftereffects in the direction of what was acquired during reach training which were on average ~-7° (t(15) = 5.24, p < .001) and was significantly smaller compared to that of SINGLE reaching (t(28) = -5.35, p < .001) but not when compared to reach aftereffects following TRACK training. The size of reduction of reach aftereffects was consistent with the extent of the interference from TRACK training as measured by the reach aftereffects produced when only that condition was performed (producing reach aftereffects of ~+9°). This CW reach aftereffect suggests that some learning remained unimpeded by the opposing perturbation during DUAL training. Overall, these findings suggest that the brain processes tracking movements somewhat independently of reaching movements and vice versa, as generalization of adaptation still tends to occur across these different types of movements.

## Discussion

Our present observations demonstrate three main points. First, we showed significant pursuit adaptation to moving targets (or tracking adaptation) following a small visuomotor perturbation to the hand-cursor. Second, while previous findings show complete interference between opposing visuomotor maps when each is associated with the same task, we found that tracking adaptation generalized to reaching movements, as reach aftereffects were present when visual feedback was withheld. These reach aftereffects following perturbed tracking movements were smaller in magnitude compared to those produced following perturbed reaching movements. Third, distinct movement types provided a strong contextual cue to the motor system when planning and producing movements for opposing visuomotor maps. This was evident in the reduction of movement errors for both reaching and tracking trials when both types of perturbed movements were experienced within the same experimental training block. Our findings support the idea that intrinsic or motor-based contextual cues, such as movement type, allow for disambiguation and retrieval of recently acquired motor memories.

### Visuomotor adaptation with tracking movements

Little is known about how the arm adapts to perturbed tracking movements towards moving targets and how this control process differs from the ubiquitous point-to-point reaching movement. Seminal work by Imamizu and colleagues showed that humans are able to adapt tracking movements in pursuit of randomly-moving targets even when the hand-cursor is perturbed by 120°[31]. Similarly, Spapé and Serrien [32] showed a reduction in pursuit errors when participants had to track a moving target with a 60°-rotated cursor. These studies suggested that humans are able to adapt pursuit movements to large perturbations of which participants were aware, although they tended to underestimate their magnitude [32]. Humans are also able to adapt pursuit movements to smaller perturbations. In one of their experimental control groups, Tong and Flanagan [21] had participants adapt to a 30°CCW rotation while tracking a target with an unpredictable trajectory and velocity across two training days. They found a significant reduction in tracking errors as well as significant savings on day two [21]. These findings demonstrate that humans are able to adapt pursuit movements, regardless of the magnitude of the perturbation or the uncertainty about the target velocity. However, since these pursuit type tasks tend to be more complex with their changing target positions, velocities, and time constraints, the distinction between point-to-point reaching and pursuit movements remains unclear. Here we show that tracking movements generalize to point-to-point reaching, suggesting overlapping neural mechanisms between these two types of movements.

Adaptation of reaching movements has also been shown to be resistant to interference by adaptation to other types of movements such as tracking movements. One key finding by Tong and Flanagan [21] was a lack of interference in the groups that experienced an opposing perturbation with a different task (i.e. reach-track-reach and reach-draw-reach interference groups). In their tasks, participants reached towards targets with a 30°CCW perturbation, followed by tracking adaptation (or “drawing” adaptation) with a 30°CW perturbation, and a re-test of the 30°CCW-perturbed reach task. They found no retrograde interference at re-test compared to a control group that simply completed reaching tasks on day 1 and day 2 under the same conditions [21]. Based on these findings, we should not expect generalization across tracking and reaching trials (i.e. reach aftereffects following tracking training). Indeed, another way to investigate whether similar mechanisms underlie reaching and tracking adaptations is to look at transfer or generalization between these two movements. Here, we found significant reach aftereffects following tracking training suggesting that some interference occurs between the opposing visuomotor maps because similar mechanisms process these different types of movements. It appears that the reduced amount of transfer following DUAL training was equivalent to the remainder of transfer from TRACK training alone. That is, the reach aftereffects we saw following DUAL training were what remained unobstructed by training concurrently with tracking movements with an opposing visuomotor mapping. Alternatively, the smaller reach aftereffect following DUAL training may simply reflect a smaller, or partial, extent of reach adaptation for this group.

It is also possible that the presence of reach aftereffects following tracking training lies in the inherent similarities between our reaching and tracking tasks movements. In Tong & Flanagan’s experiments [21], the reaching and tracking tasks involved very different target dynamics that made it difficult to tease out exactly whether movement type was the key distinction between tasks. While their reach task was a typical out-and-back movement, the tracking task had a target that moved unpredictably (i.e. a pattern using a sum of 5 sine wave functions) with varying velocity with a large standard deviation. Additionally, their tracking task had no penalty for slow movements, which meant that movements could extend longer than their 35-second trial window [21]. All these differences between the reach and the tracking tasks suggest that the movement was not exploring the same space (i.e. not only requiring different movements but also the complete context altogether). In order to isolate the role of different types of movement in visuomotor adaptation, we designed a tracking adaptation task that resembles reach adaptation using a small rotation, a small subset of non-random paths, a constant velocity for all target movement, and the same workspace that requires a similar outward movement for both types of movements. Critically, the same desired trajectory is required across reaching and tracking trials. This ensures that the movement type alone allows for the facilitation of dual adaptation.

Yet another explanation for the transfer of learning between tracking and reaching tasks may be due to the nature of the tracking task itself. In tracking trials, corrections are readily available throughout the movement thereby making it redundant to store a stronger representation of the internal model for that visuomotor map. Similar findings by Ikegami and colleagues show that adapted rhythmic movements show little transfer to reaching movements [33]. Likewise, findings from Morehead et al. [22] show smaller reach aftereffects following adaptation of pursuit movements to randomly moving targets compared to those following discrete reaching towards static targets. Thus, it is possible that when errors can be corrected in flight as in tracking movements, the motor system is less likely to rely on an internal model. Since we found significant reach aftereffects following rotated tracking training (i.e. generalization), tracking adaptation might not be accomplished by online corrections alone, but through a combination of processes which may include the formation of a learned internal model.

One of the main characteristics that distinguishes tracking and reaching from one another is the speed of these hand movements. Thus, differences in speed may be contributing to the efficacy of movement types as contextual cues for dual learning. For instance, in one prism adaptation task, Kitazawa and colleagues found smaller reach aftereffects when the movement times during prism exposure and prism removal phases greatly differed, with a small significant difference when the movement times were 2.5 times different; larger differences were found when movement times differed tenfold [34]. Conversely, Goodbody and Wolpert found that for force-field adaptation tasks, learning tends to generalize between different speeds [35]. For our tracking task, in order to induce optimal tracking movements, we specifically implemented a fixed target speed that participants can easily match, while reaching speed was left unrestricted. Thus, by definition, the two movement types had different speeds. Indeed, tracking took slightly more than twice as long as the ballistic reaches, and thus, we expect that some of the differences in the reach aftereffects and the learning rate during DUAL training could be related to differences in movement duration. In either case, movement speed or movement type could both provide a sufficient context to allow for dual adaptation.

Theoretical models might suggest that differences in the costs associated with these different movements, such as speed, are integrated into the learned internal models given what type of information is coded during the planning stage. In one model called the Modular Selection and Identification for Control (MOSAIC) theory, humans are thought to be able to flexibly adapt their movements and choose the right compensatory action given a variable environment with the help of modules consisting of a forward model, inverse model, and responsibility predictor [36,37]. In this model, both feedforward and feedback information (which include velocity) are combined to select the appropriate controller for the context. Thus, it may be possible that for different velocities (and thus, distinct contexts), different model pairs are selected to produce the correct compensatory action. Thus, it may be possible that both different arm movements and divergent target velocities jointly facilitate dual adaptation. Future studies should explore whether movement velocity alone can act as a strong contextual cue for facilitating dual learning.

Lastly, although we do not record eye movements during the experiments, it is likely that participants remained fixated or in pursuit of the target due to the absence of other visual stimuli in the task display, as typically seen in reaching tasks [38–40]. Indeed, recent work suggests that for 30° rotations, gaze remains fixated on the target until movement is completed [38,41,42]. Thus, reaches and tracking movements likely were associated with different eye movements (saccades and smooth pursuits) and it is possible that these different eye movements also contributed as intrinsic cues to facilitate dual adaptation.

### Explicit strategies

What do the reach aftereffects following SINGLE and DUAL tracking training signify? While it was initially thought that motor adaptation was predominantly an implicit process, more recent work suggests that early stages of motor learning may reflect explicit strategies when compensating visuomotor rotations [27,43]. This could explain the reduced learning rate during DUAL training compared to that of the SINGLE training since it may be more difficult to develop an explicit aiming strategy without sufficient contextual cues. We speculate that the slower learning rate during DUAL training reflects a slower development of an explicit strategy or possibly merely reflect only the contribution of the implicit learning as a function of the different movement types. In all three groups, our participants often had no awareness of the perturbation or at the least, were unable explain the nature of the rotations, suggesting that they were not likely forming compensatory strategies. Nonetheless, we do see significant reach aftereffects in all three groups that are consistent with implicit learning [28]. The smaller reach aftereffects associated when only training with a moving target and or when reaching and tracking in the same training blocks likely reflects the extent of the transfer (or its inverse, interference) of implicit learning. Thus, this may suggest that the contextual cue of movement types is sufficient to allow for concurrent implicit learning of opposing perturbations and perhaps even some contribution of explicit learning during training. Still, it remains unknown to what extent explicit processes contribute to dual adaptation, laying fertile ground for future investigations.

### Incomplete transfer and partial adaptation

When the motor systems constantly experiences opposing error signals, it is not surprising that it learns less as a result [44]. However, as we have shown, when a context is associated to an error signal, interference may be attenuated. How then does the motor system utilize error to plan and control movement for each context? Nozaki and Scott [15] proposed a multi-compartmental state-space model to explain how opposing force fields can be learned simultaneously when each are associated with a different context for overlapping unimanual and bimanual tasks. In their model, a global update rule is applied to update each component in the system [15]. Their model was able to explain why they found significant yet only partial dual learning between unimanual and bimanual movements in their combined learning condition [16]. These findings can also explain the efficacy of learning across our two behavioural contexts: reaching and tracking movements. Much like bimanual movements requiring complex bihemispheric interactions for planning [45,46], tracking movements may require a temporal matching component that engages distinct neural substrates that distinguish them from point-to-point reaching movements. This suggests that both unimanual reaching and tracking compartments of the system use common error information to update the internal state of the system and thus, involve overlapping neural processes.

A related finding is that dual adaptation tends to be partial when the targets are identical across visuomotor maps. In our present experiments, we observed a significant reduction in reach errors over training that, nonetheless, does not reach baseline levels. When globally comparing between our past experiments which looked at cues such as hand posture, body posture, reach skew (via an obstacle avoidance), and the present experiments, we find that the extent of DUAL rotation learning ranges from around a third to a half compared to SINGLE rotation learning [6,13]. In only one of our experiments, we found near-complete learning—the case for dual adaptation when each perturbation was associated with a distinct target workspace location [6]. This is expected given very little generalization tends to occurs for divergent movement directions [47]. Thus, by using the same target set for all conditions and keeping reach direction constant, any effect would be likely due to the efficacy of the cue itself.

## Conclusions

Perturbed tracking of moving targets produced significant reach aftereffects that are smaller than those produced for static targets. This suggests that visually-guided changes in tracking movements generalize to reaching movements. Since this generalization is not complete, we tested whether this distinction in movement type can be used to contextualize and thus, facilitate dual adaptation to opposing visuomotor rotations. We found further evidence that motor-based cues such as movement type provide a strong context for the motor system when they are the only cues provided to dissociate between two opposing rotations, and thus, two different visuomotor maps, even when desired cursor trajectories are identical.
